# Assessment of renal allograft function using amide proton transfer imaging

**DOI:** 10.3389/fmed.2025.1612028

**Published:** 2025-08-29

**Authors:** Yaping Ge, Jian Du, Haichao Cheng, Wei Wang

**Affiliations:** ^1^Graduate School of Dalian Medical University, Dalian, Liaoning, China; ^2^Department of Radiology, The First Affiliated Hospital of Shandong First Medical University, Jinan, Shandong, China; ^3^Department of Urology, The First Affiliated Hospital of Shandong First Medical University, Jinan, Shandong, China; ^4^Department of Radiology, Affiliated Hospital of Yangzhou University, Yangzhou, Jiangsu, China

**Keywords:** amide proton transfer, renal allograft, functional magnetic resonance imaging, renal function, estimated glomerular filtration rate

## Abstract

**Objective:**

This study aimed to investigate the ability of amide proton transfer (APT) imaging to assess the function of transplanted kidneys.

**Methods:**

Between October 2023 and July 2024, a total of 44 renal allograft recipients were recruited prospectively, who underwent renal APT imaging with a 3.0 T system 2–3 weeks after transplantation. Recipients were divided into two groups according to the estimated glomerular filtration rate (eGFR): group A, eGFR < 60 mL/min/1.73 m^2^; and group B, eGFR ≥60 mL/min/1.73 m^2^. The relationships between cortical and medullary APT values and allograft function were assessed using the Spearman's correlation coefficient. The paired sample *t*-test was used to compare cortical and medullary APT values. APT values in groups A and B were compared using the Student's *t*-test or Mann–Whitney *U*-test. Receiver operating characteristic curves were generated to assess the ability of cortical and medullary APT values to diagnose impaired allograft function.

**Results:**

Two physicians calculated APT values independently and with good agreement, as indicated by an intraclass correlation coefficient > 0.75. The renal cortical and medullary APT values in group A (2.61% ± 0.51% and 2.11% ± 0.37%, respectively) were significantly higher than those in group B (1.77% ± 0.13% and 1.79% ± 0.29%, respectively) (*P* < 0.05). APT values in the renal cortex and medulla were negatively correlated with eGFR (*r* = −0.8551 and *r* = −0.5163, respectively; *P* < 0.01). In group A, cortical APT values were higher than medullary APT values (*P* < 0.05). Cortical and medullary APT values demonstrated a good ability to diagnose impaired renal allograft function. In group A, mean cortical APT values were higher in patients whose renal function did not recover (3.10% ± 0.54%) than in those with stable allograft function 6 months after transplantation (2.50% ± 0.44%) (*P* < 0.05).

**Conclusion:**

APT imaging is a promising technique for non-invasive functional assessment of renal allografts.

## 1 Introduction

Kidney transplantation has become the primary treatment choice for many patients with end-stage renal disease, as it can improve quality of life and reduce morbidity and mortality (1). Recipients of kidney transplants require close monitoring to allow early identification of allograft dysfunction and the initiation of appropriate treatment in order to prevent serious consequences, especially in the early stages after transplantation (2). Renal allograft function is monitored mainly by measuring serum creatinine levels, ultrasonography, and examining renal biopsies. However, the serum creatinine and ultrasound methods are insensitive and non-specific, and renal graft biopsy, although the gold standard for evaluating renal graft impairment, is an invasive procedure and therefore may result in complications such as bleeding, infection, and arteriovenous fistula formation (3). In addition, sampling limitations are likely to cause deviations in the results of renal biopsies (4). It is therefore important to develop an accurate, safe, and rapid method of monitoring renal allograft function to allow timely clinical treatment and thus prevent or delay irreversible damage to the transplanted kidney.

In recent years, non-invasive functional magnetic resonance imaging (MRI) techniques such as arterial spin labeling, diffusion-weighted imaging, diffusion tensor imaging, intravoxel incoherent motion imaging, and blood oxygen level-dependent imaging have been increasingly used in clinical research into renal transplantation and injury. These methods quantitatively evaluate renal perfusion, diffusion, oxygenation, and metabolism and have the potential to dynamically monitor microstructural and functional changes in transplanted kidneys (5–8).

Amide proton transfer (APT) imaging is a molecular MRI technology and a form of chemical exchange saturated transfer (CEST) imaging. It is sensitive to the concentration of mobile proteins and peptides in tissues (9) and to pH values that affect the rate of chemical exchange (10). APT imaging can indirectly reflect metabolic changes and pathophysiological information in living cells. Currently, APT imaging is widely performed in the nervous system and in tumor lesions (11–17), and recent studies have confirmed its ability to detect kidney damage in patients with chronic kidney disease and renal fibrosis (18, 19). However, the use of APT imaging to monitor renal function after transplantation has not yet been reported. This study aimed to investigate the value of APT imaging in assessing renal function in patients with kidney transplants and the correlation between APT values and the estimated glomerular filtration rate (eGFR).

## 2 Materials and methods

### 2.1 Patients

This study was approved by the Institutional Ethical Review Committee in accordance with institutional guidelines. Written informed consent was obtained from all participants. This prospective study recruited patients between October 2023 and July 2024. The inclusion criteria were as follows: (1) primary kidney transplant, (2) an interval of 2–3 weeks between transplantation and MRI, and (3) no contraindications for MRI. Ten patients were excluded because of the following reasons: (1) diagnosis of combined lung and heart disease (*n* = 2); (2) routine ultrasonography before MRI indicated hydronephrosis (*n* = 2), perirenal effusion (*n* = 1), ureteral obstruction (*n* = 1), or renal artery stenosis (*n* = 1); and (3) poor image quality (*n* = 3), such as the presence of breathing artifacts, motion artifacts, or drainage tube interference artifacts.

Venous blood was collected from all patients on the day of MRI and used to measure serum creatinine levels and calculate the eGFR using the Modification of Diet in Renal Disease formula (20). Patients were divided into two groups based on eGFR: group A, impaired allograft function (eGFR < 60 mL/min/1.73 m^2^); and group B, good allograft function (eGFR ≥ 60 mL/min/1.73 m^2^). The renal function of patients was determined by measuring the eGFR once a week for 1 year after transplantation and at least every 3 months thereafter. All patients were followed up for at least 6 months.

### 2.2 MRI

All patients underwent MRI in the supine position using a Discovery MR750w 3.0 T system (GE HealthCare, Chicago, IL, USA) and an eight-channel phased-array body coil. First, T2-weighted image (T2WI) scanning of coronal fast spin echo sequences was performed, followed by T1WI and T2WI scanning of the axial position. Finally, APT sequence scanning was performed. The APT sequence selected the renal allograft to display the largest layer of coronal single-layer scanning. APT scanning parameters were as follows: coronal position; repetition time, 3,000 ms; echo time, 37.7 ms; thickness, 5 mm; spacing, 0; scanning field of view, 240 × 240 mm^2^; matrix (frequency × phase), 128 × 128; frequency direction, S/I; slice, 1; and scanning time, 2 min 41 s.

### 2.3 MRI image analysis

All images were transferred to Advantage Workstation version 4.6 (GE Medical Systems). APT data were processed using vendor-provided Function tool APT software (GE HealthCare). Two physicians with 10 and 7 years of experience with abdominal MRI and who were blinded to the clinical data independently selected the regions of interest (ROIs), referring to the anatomical diagram of the transplanted kidney. Five ellipsoid ROIs of 8–13 mm^2^ were placed in the renal cortex, and ROIs of 15–25 mm^2^ were placed to cover the renal medulla. The mean APT value for each ROI was obtained. The average APT value of the two radiologists was used as the final result for statistical analysis.

### 2.4 Statistical analysis

All statistical analyses were performed using SPSS version 26.0 (IBM Corporation, Armonk, NY, USA) and GraphPad Prism version 10.4.0 (GraphPad Software, La Jolla, CA, USA). The normality of the data was determined using the Shapiro–Wilk test. Normally distributed data are described using the mean ± standard deviation, whereas non-normally distributed data are described using the median (interquartile range). Categorical variables were compared using the chi-squared test. The intraclass correlation coefficient (ICC) was calculated to determine interobserver variation. The correlation between APT values and the eGFR was assessed using Spearman's rank correlation coefficient. According to the normality of data distribution, the independent-sample *t*-test or Mann–Whitney *U*-test was used to compare continuous variables. To account for multiple comparisons, we applied the Bonferroni correction. The paired sample *t*-test was used to compare the differences in APT values between the renal cortex and medulla. The ability of APT values to differentiate between impaired and good renal allograft function was assessed by generating receiver operating characteristic (ROC) curves and using these to calculate the area under the curve (AUC), sensitivity, specificity, and accuracy. The DeLong test was used to compare ROC curves. Statistical significance was set at *P* < 0.05. *Post-hoc* power calculations were performed using G^*^Power version 3.1.9.7(Heinrich Heine Universitat Dusseldorf, Dusseldorf, Germany).

## 3 Results

### 3.1 Patient characteristics

The 44 patients with kidney transplants included in this study had a mean age of 42.12 ± 11.05 years (range, 17–62 years); 30 were male and 14 were female. Of these, 28 (63.6%) had impaired allograft function and were allocated to group A, and 16 (36.4%) had good allograft function and were allocated to group B. The clinical characteristics of the participants are presented in Table 1.

**Table 1:** Characteristics of participants.

**Clinical characteristics**	**Group A (*n* = 28)**	**Group B (*n* = 16)**	***P***
Age (years)	42.5 (35.3, 51.8)	38.5 (36.25, 46.25)	0.479
Sex, no (%)			0.951
Female	9 (32.1%)	5 (31.2%)	
Male	19 (67.9%)	11 (68.8%)	
Time after transplantation (days)	10 (8, 18)	11 (7.5, 12)	0.556
Cold ischemia time (h)	4 (4.0, 5.0)	4 (3.3, 4.8)	0.266
eGFR(ml/min/1.73 m^2^)	35.4 ± 11.2	73.2 (67.6, 103.1)	< 0.001
Scr (umol/L)	205.5 (156.8, 243.8)	95.5 (68.5, 130.0)	< 0.001
CysC (mg/L)	2.5 ± 0.7	1.3 ± 0.3	< 0.001

eGFR, estimated glomerular filtration rate; Scr, serum creatinine; CysC, Cystatin C.

### 3.2 Inter-observer agreement

The ICC, calculated to determine the agreement of the two physicians who measured APT values in the cortex and medulla of the transplanted kidney, was > 0.75, indicating good reliability (Table 2).

**Table 2:** Inter-observer agreement of multiple parameters.

**Patient group**	**No**	**Region**	**APT (%)**		**ICC**
			**Physician 1**	**Physician 2**	
Group A	28	Cortex	2.66 ± 0.55	2.55 ± 0.48	0.93
		Medulla	2.12 ± 0.41	2.11 ± 0.36	0.90
Group B	16	Cortex	1.79 ± 0.15	1.76 ± 0.14	0.77
		Medulla	1.77 ± 0.31	1.80 ± 0.28	0.89

ICC, intraclass correlation coefficient.

### 3.3 Comparison of APT values between the two groups

**Figure 1:**
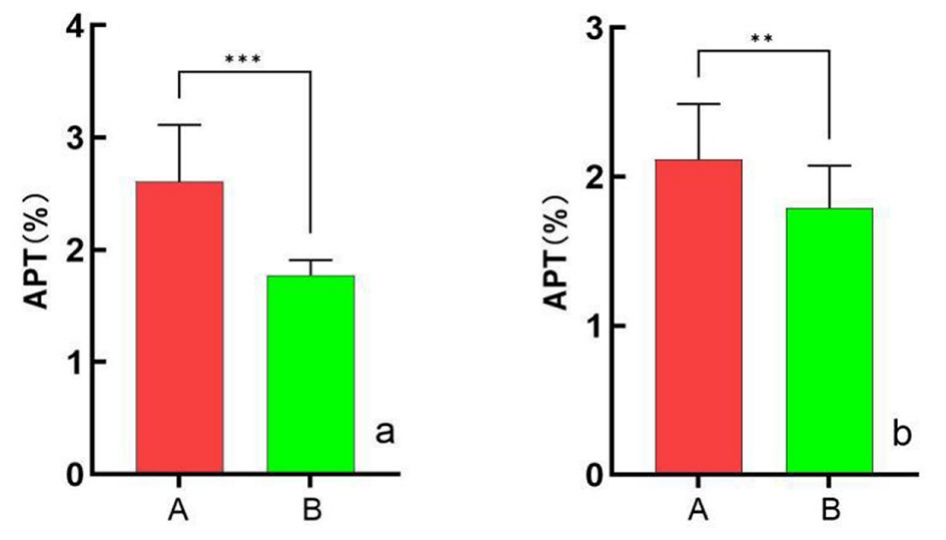
Comparison of cortical **(a)** and medullary **(b)** APT values in groups A and B. The cortical APT values in group A and B are 2.61% ± 0.51%, 1.77% ± 0.13%. The medullary APT values in group A and B are 2.12% ± 0.37%, 1.79% ± 0.29%. Both the cortical and medullary APT values were higher in group A than in group B (***p < 0.001, **p < 0.05, respectively). APT, amide proton transfer.

**Figure 2:**
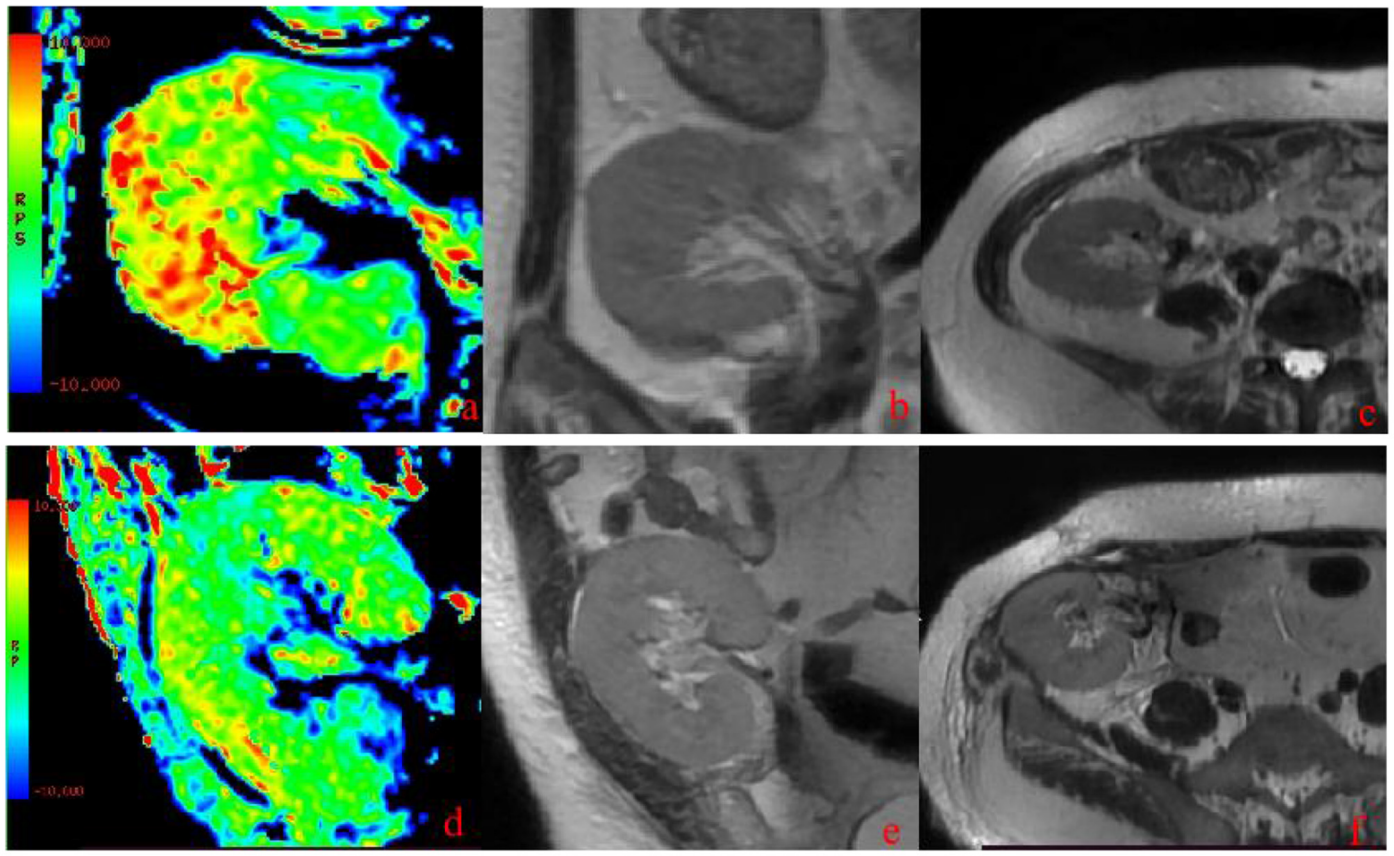
Representative APT images. **(a–c)** Images of a 38-year-old woman with impaired renal allograft function 14 days after transplantation (group A; eGFR = 50.35 mL/min/1.73 m^2^). **(a)** APT image shows renal allograft in the right iliac fossa. The cortical and medullary APT values were 2.39% and 2.08%, respectively. **(b, c)** T2-weighted images. **(d–f)** Images of a 34-year-old man with good renal allograft function 19 days after transplantation (group B; eGFR = 72.09 mL/min/1.73 m^2^). **(d)** APT image shows renal allograft in the right iliac fossa. The cortical and medullary APT values were 1.78% and 1.37%, respectively. **(e, f)** T2-weighted images. APT, amide proton transfer; eGFR, estimated glomerular filtration rate.

The APT values of the cortex and medulla were significantly higher in group A than in group B (*P* < 0.05) (Figure 1). Representative images are shown in Figure 2. The cortical APT values showed a gradually increasing trend compared with the medullary APT values in group A (*P* < 0.05). In group B, the cortical APT values were slightly lower than the medullary APT values (*P* > 0.05) (Table 3).

**Table 3:** Comparison of APT values in the two groups.

**Patient group**	**Cortex APT (%)**	**Medulla APT (%)**	***P***
Group A	2.61 ± 0.51	2.12 ± 0.37	< 0.05
Group B	1.77 ± 0.13	1.79 ± 0.29	>0.05

### 3.4 Correlation of APT values with eGFR

**Figure 3:**
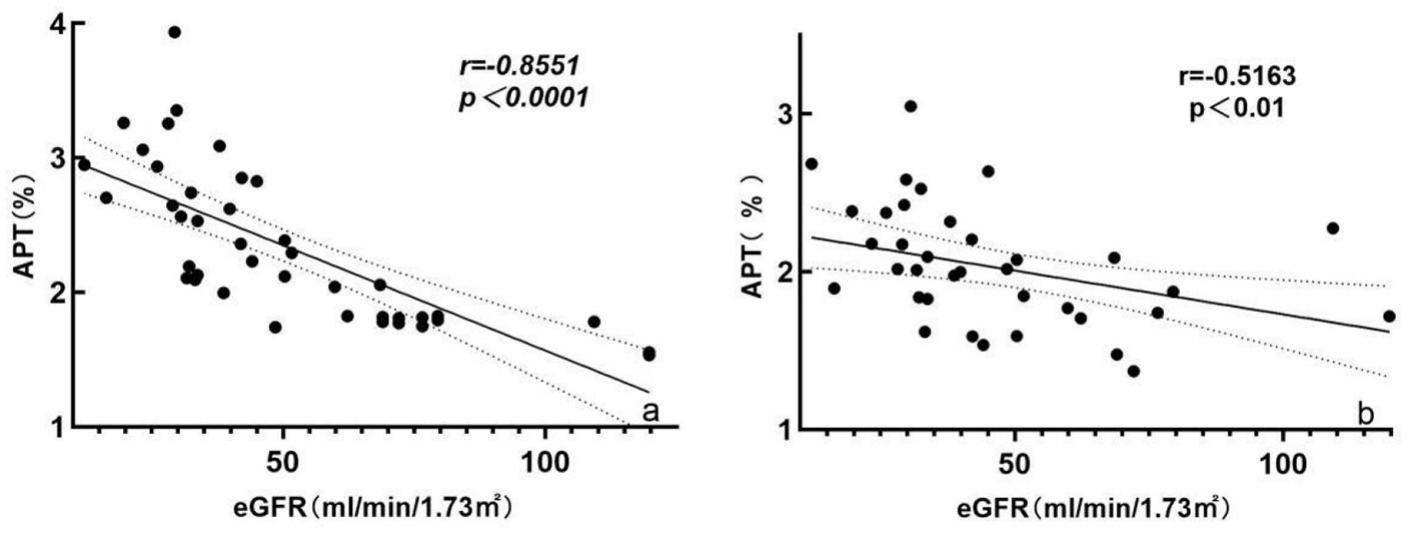
Scatterplot showing the correlations of cortical **(a)** and medullary **(b)** APT values with the eGFR. Both the cortical (*r* = −0.8551, *P* < 0.0001) and medullary (*r* = −0.5163, *P* < 0.01) APT values were negatively correlated with the eGFR. APT, amide proton transfer; eGFR, estimated glomerular filtration rate.

The Spearman's correlation analysis showed that cortical APT values were negatively correlated with the eGFR (*r* = −0.8551, *P* < 0.0001). Medullary APT values were also negatively correlated with the eGFR (*r* = −0.5163, *P* < 0.01) (Figure 3).

### 3.5 Diagnostic ability of APT values

**Figure 4:**
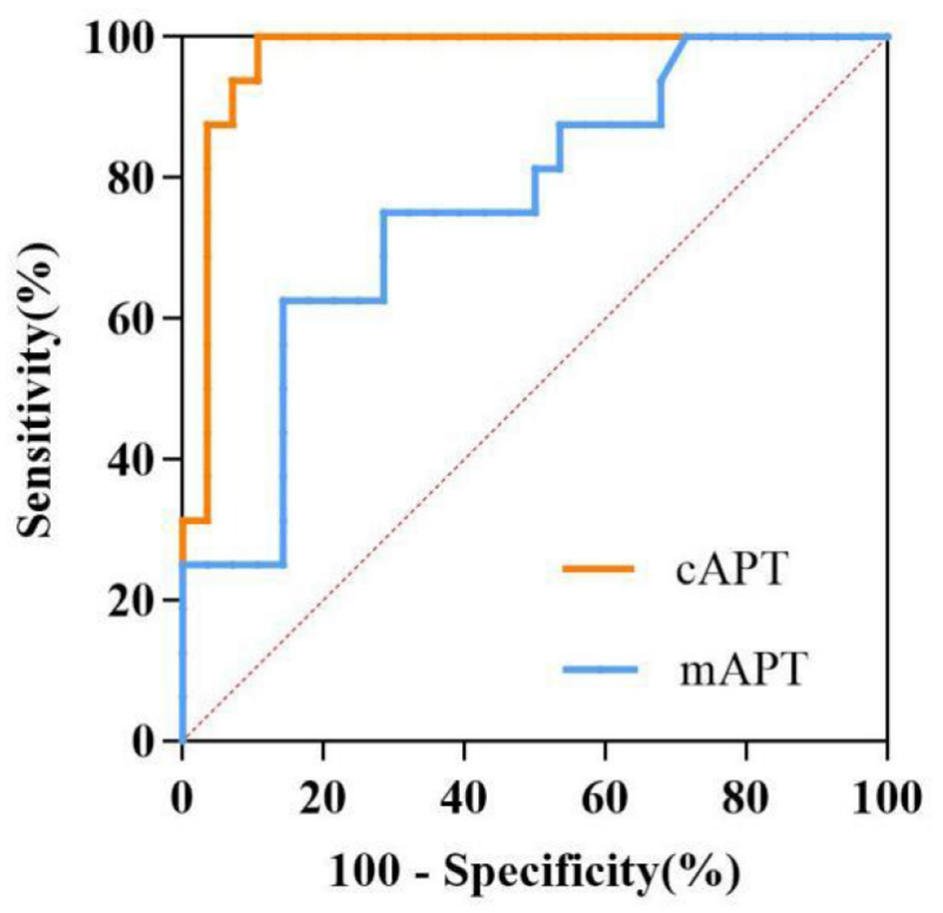
ROC curves of the ability of cAPT and mAPT values to differentiate between groups A and B. The areas under the curves of the cAPT and mAPT are 0.969, 0.760, respectively. cAPT, cortex amide proton transfer; mAPT, medulla amide proton transfer; ROC, receiver operating characteristic.

The ability of cortical and medullary APT values to distinguish between groups A and B is shown in Table 4. The AUC of the cortical APT values was significantly greater than that of the medullary APT values (*P* < 0.05) (Figure 4).

**Table 4:** Ability of APT values to determine renal allograft function.

**APT (%)**	**AUC**	**95% CI**	**Optimal cutoff value**	**Sensitivity (%)**	**Specificity (%)**
Cortex	0.969	0.918–1.000	2.074	89.3	100.0
Medulla	0.760	0.613–0.907	1.767	85.7	62.5

All patients in group B and 23 of the 28 patients in group A had stable allograft function 6 months after transplantation. The renal function of the five remaining patients in group A did not recover due to acute tubular necrosis (two patients), deterioration of allograft function following surgery (one patient), permanent hemodialysis (one patient), and thrombotic microangiopathy (one patient). In group A, cortical APT values were higher in patients with unrecovered renal function than in patients with stable renal function at 6 months, whereas medullary APT values and eGFR did not differ significantly (Table 5).

**Table 5:** Comparison of group A eGFR and APT values according to allograft function 6 months after transplantation.

**Parameter**	**Stable allograft function (*n* = 23)**	**Allograft failure (*n* = 5)**	***P***
eGFR (mL/min/1.73 m^2^)	37.05 ± 10.45	28.01 ± 12.71	>0.05
Cortex APT (%)	2.50 ± 0.44	3.10 ± 0.54	< 0.05
Medulla APT (%)	2.06 ± 0.36	2.39 ± 0.34	>0.05

APT, amide proton transfer; eGFR, estimated glomerular filtration rate.

## 4 Discussion

In this study, we performed APT imaging on renal transplant recipients during the early post-transplantation period. This study demonstrated the ability of APT values to quantitatively evaluate renal allograft function and showed a correlation between cortical APT values and eGFR.

APT imaging is a molecular MRI technique derived from CEST imaging (21) that can provide insights into the physicochemical properties of the tissue, capture the exchange rate between amide and water protons, reflect the concentration of mobile macromolecules such as proteins and peptides (22), and is affected by factors such as pH (23).

APT imaging has been performed on kidneys to evaluate functional damage in chronic kidney diseases and renal fibrosis (18, 19, 24). Consistent with findings of previous studies, the present study showed that APT values of the renal cortex and medulla were negatively correlated with the eGFR. This suggests that the APT values of the renal cortex and medulla reflect the function of the transplanted kidney and may have some value in the diagnosis of renal allograft dysfunction. A previous study (18) has shown that cortical APT values are higher than medullary APT values. This may be due to the fact that the renal cortex is composed of renal corpuscles and tubules and is rich in blood vessels, whereas the renal medulla is mainly composed of collecting ducts with a low blood supply. The renal cortex accounts for 94% of renal perfusion, significantly higher compared with the medulla. Therefore, the rate of protein exchange in the renal cortex can be significantly higher than that in the medulla. Previous research (25) has also shown a gradual decrease in renal pH from the cortex to the medulla. The lower the pH of the tissue, the lower the APT values. The present study found that cortical APT values were higher than medullary APT values in group A, consistent with previous findings. In group B, the cortical APT values were lower than the medullary APT values, although the difference did not reach statistical significance. Previous chronic kidney disease and renal fibrosis research has shown that cortical and medullary APT values gradually increase with the severity of renal impairment. This increase is due to the excessive deposition of extracellular matrix, namely collagen, non-collagenous glycoproteins, and proteoglycans, during tissue fibrosis. The kidney plays an important role in the maintenance of electrolyte homeostasis and acid–base balance. A recent study in mice showed that the renal APT value following acute kidney injury was higher than that of normal kidneys (26). An animal study (27) revealed that a decrease in or obstruction of blood perfusion can damage functional nephrons, resulting in impaired urinary acidification, which increases renal pH and the exchange rate of amide and water protons. There are many causes of allograft renal dysfunction early after transplantation, including endothelial cell ischemia, which can lead to cell damage and swelling, impaired blood flow, and reperfusion, mainly representing ischemia-reperfusion injury, glomerular necrosis, or necrosis of renal tubular epithelial cells due to decreased blood perfusion (28). In clinical practice, the acute rejection (AR) and acute tubular necrosis (ATN) are the main causes of early graft dysfunction (8, 29, 30). AR mainly occurs in glomerular lesions, such as glomerular sclerosis or fibrinoid necrosis, and even thrombosis that blocks the renal arterioles, thereby reducing renal blood flow. ATN is characterized by degeneration and necrosis of renal tubular epithelial cells, accompanied by interstitial edema and inflammatory cell infiltration. These pathological changes lead to compression of renal microvasculature and subsequent reduction in renal perfusion. The resultant hypoperfusion damages functional nephrons, leading to altered renal pH and enhanced exchange rates between amide and water protons. Consistent with this pathophysiological mechanism, our study demonstrated a significant negative correlation between cortical/medullary APT values and eGFR. Renal allograft dysfunction decreases Na+/K+ ATP activity, destroying the acid–base balance. This may alter renal pH to some extent, leading to an increase in the exchange rate between amide and water protons and therefore increasing APT values. Previous research (31) used CEST MRI to detect changes in renal pH and showed a good correlation between pH and blood urea nitrogen levels. This is consistent with the results of the present study, which demonstrated a good correlation between APT values and eGFR. The present study also showed that cortical and medullary APT values were higher in group A than in group B and could be used to distinguish between the groups.

In group A in the present study, cortical APT values were higher in patients whose renal function did not recover than in patients who had stable renal function 6 months after transplantation, whereas eGFR and medullary APT values did not differ significantly. This may be because the early stages of renal allograft dysfunction affect renal parenchymal perfusion but do not cause significant changes in glomerular filtration. However, as renal dysfunction progresses, the impact on renal function will gradually become evident. This suggests that cortical APT values may be a more sensitive measure than eGFR for early renal allograft dysfunction. Our findings suggest that APT imaging can sensitively detect pathological changes in the renal allograft microstructure, indicating that the non-invasive assessment of renal function has great clinical significance for disease diagnosis and prognosis.

There are some limitations to our study. First, the transplanted kidneys, including the functionally impaired allografts, were not subjected to pathological analyses, and therefore the APT values could not be analyzed in the context of the pathological findings. We will include pathological data in our future research. Second, the study had a small sample size, particularly in terms of patients with good allograft function, which might have introduced bias. Thus, we will increase the patients accrual in our hospital and prolong follow-up period. Besides, a multicenter collaboration involving to enhance patient sample size in our future research. Third, APT imaging selects the maximum cross-sectional area of the kidney instead of the entire kidney, which may affect the accuracy of the test results. Four, we have not yet perform direct comparisons between APT MRI and other functional MRI methods with the same cohort. In the future, we will study multiple functional sequences together on the kidney transplant patients to explore their greater clinical value.

In conclusion, the APT values of the renal cortex differed significantly between allograft recipients with impaired renal function and those with good renal function. The high correlation between the APT values of the renal cortex and allograft function highlights the potential use of APT imaging for the non-invasive functional assessment of transplanted kidneys.

## Data Availability

The raw data supporting the conclusions of this article will be made available by the authors, without undue reservation.
